# The Prognostic Value of BRCA1 mRNA Expression Levels Following Neoadjuvant Chemotherapy in Breast Cancer

**DOI:** 10.1371/journal.pone.0009499

**Published:** 2010-03-03

**Authors:** Mireia Margeli, Beatriz Cirauqui, Eva Castella, Gustavo Tapia, Carlota Costa, Ana Gimenez-Capitan, Agusti Barnadas, Maria Sanchez Ronco, Susana Benlloch, Miquel Taron, Rafael Rosell

**Affiliations:** 1 Medical Oncology Service, Department of Medicine, Catalan Institute of Oncology, Hospital Germans Trias i Pujol and Autonomous University of Barcelona, Badalona, Spain; 2 Pathology Service, Hospital Germans Trias i Pujol, Badalona, Spain; 3 Pangaea Biotech, SA, USP Dexeus University Institute, Barcelona, Spain; 4 Medical Oncology Department, Hospital Santa Creu i Sant Pau, Barcelona, Spain; 5 Alcalá de Henares University, Madrid, Spain; University of Barcelona, Spain

## Abstract

**Background:**

A fraction of sporadic breast cancers has low BRCA1 expression. BRCA1 mutation carriers are more likely to achieve a pathological complete response with DNA-damage-based chemotherapy compared to non-mutation carriers. Furthermore, sporadic ovarian cancer patients with low levels of BRCA1 mRNA have longer survival following platinum-based chemotherapy than patients with high levels of BRCA1 mRNA.

**Methodology/Principal Findings:**

Tumor biopsies were obtained from 86 breast cancer patients who were candidates for neoadjuvant chemotherapy, treated with four cycles of neoadjuvant fluorouracil, epirubicin and cyclophosphamide. Estrogen receptor (ER), progesterone receptor (PR), HER2, cytokeratin 5/6 and vimentin were examined by tissue microarray. HER2 were also assessed by chromogenic *in situ* hybridization, and BRCA1 mRNA was analyzed in a subset of 41 patients for whom sufficient tumor tissue was available by real-time quantitative PCR. Median time to progression was 42 months and overall survival was 55 months. In the multivariate analysis for time to progression and overall survival for 41 patients in whom BRCA1 could be assessed, low levels of BRCA1 mRNA, positive PR and negative lymph node involvement predicted a significantly lower risk of relapse, low levels of BRCA1 mRNA and positive PR were the only variables associated with significantly longer survival.

**Conclusions/Significance:**

We provide evidence for a major role for BRCA1 mRNA expression as a marker of time to progression and overall survival in sporadic breast cancers treated with anthracycline-based chemotherapy. These findings can be useful for customizing chemotherapy.

## Introduction

Breast cancer is the most common cancer in women worldwide, comprising 23% of all cancers, with more than 1 million new cases per year[1]. According to the American Cancer Society, breast cancer death rates have been dropping steadily since 1990 because of earlier detection and better treatments. Nevertheless, new strategies are necessary to improve survival of breast cancer patients, especially in those with advanced disease.

Neoadjuvant chemotherapy is standard therapy for patients with locally advanced breast cancer and is increasingly used for early-stage operable disease. The response of breast cancer to neoadjuvant chemotherapy is correlated with survival [2]; patients who obtain the greatest survival advantage from neoadjuvant chemotherapy are those who attain complete response of their primary tumor [3].

Microarray analysis has identified breast cancer subtypes with distinct gene expression profiles [4], [5]. These subtypes have been correlated with clinical outcome, and the impact of subtype on response to neoadjuvant chemotherapy has been evaluated in different series [6]. Easily assessable markers can be used to approximate breast cancer subtype. Specifically, using the estrogen receptor (ER), progesterone receptor (PR), and HER2 status of a tumor, breast subtype can be approximated as follows: luminal A (ER+ or PR+ and HER2–), luminal B (ER+ or PR+ and HER2+), HER2+/ER- (ER– and PR– and HER2+), and basal-like (ER– and PR– and HER2–) [7], [8].

Basal-like tumors frequently express ‘basal’ cytokeratins (CKs) such as CK-5/6, CK-14 and CK-17 [9]. Vimentin expression, a rather rare occurrence in invasive breast cancer, is associated with high tumor invasiveness [10] and with *in vitro* chemosensitivity[11].

Breast cancer susceptibility gene 1 (BRCA1) plays an important role in DNA repair via transcription-coupled nucleotide excision repair [12]. BRCA1 encodes a multifactorial protein that is implicated in DNA repair, cell cycle checkpoint control, transcriptional regulation, and ubiquitination. BRCA1 methylation and abrogation of BRCA1 mRNA have been reported in sporadic breast cancers [13]. Somatic BRCA1 mutations are rarely observed in sporadic breast cancer; however epigenetic downregulation of BRCA1 has been reported in approximately 30% of sporadic breast cancers and 70% of ovarian cancers [14]. BRCA1 expression can modulate cellular response to chemotherapy. Preclinical breast cancer studies suggest a role for BRCA1 in predicting response to DNA-damaging agents and taxane-based chemotherapy. Decreased BRCA1 mRNA expression in breast cancer cell lines, as determined by real-time quantitative polymerase chain reaction (RT-QPCR), enhances cisplatin sensitivity but leads to resistance to paclitaxel and vinorelbine via defective apoptotic response to these drugs, while the opposite phenomenon is observed in the presence of normal or high levels of BRCA1 [15]. In some sporadic breast cancers, the poor outcome associated with BRCA1 methylation and low expression levels could be explained by MYC amplification [16]. Furthermore, several retrospective breast cancer studies have confirmed that carriers of BRCA1 mutations gained more benefit from DNA-damage-based chemotherapy [17]. Low levels of BRCA1 mRNA were associated with longer survival in a retrospective cohort of lung cancer patients following cisplatin gemcitabine [18] and in two retrospective cohorts of ovarian cancer patients treated with platinum-based chemotherapy [19]. All these studies suggest that not only BRCA1 mutations but also reduced expression levels of BRCA1 mRNA could predict a benefit from DNA-damage-based chemotherapy. In clinical practice, fresh tumor tissue is not always available, and the recovery of mRNA from paraffin-embedded tissue is crucial. RT-QPCR permits quantitative and accurate measurement of gene mRNA expression [20].

In a retrospective series of 86 breast cancer patients treated with neoadjuvant fluorouracil, epirubicin and cyclophosphamide (FEC), we evaluated response, time to progression (TTP) and overall survival (OS) according to the simplified classification of breast cancer subtypes based on ER, PR and HER2. In addition, we examined CK5/6, vimentin and HER2 by immunohistochemistry; and HER2 by chromogenic *in situ* hybridization (CISH). Finally, in 41 patients for whom sufficient tumor tissue was available, intratumoral BRCA1 mRNA levels were assessed by RT-QPCR. All findings were correlated with response, TTP and OS.

## Results

### Patient Characteristics

Table 1 shows patient characteristics for all patients (86 patients), for the 41 patients in whom BRCA1 mRNA levels were assessed, and for the 45 patients in whom BRCA1 mRNA levels were not assessed. Patient characteristics were similar across the groups. Median age at diagnosis was 54 years (range, 31-79). Fifty patients (58%) were postmenopausal. At diagnosis, 24 patients (28%) were stage II, 62 patients (72%) were stage III. All 86 patients were considered to be candidates for primary therapy according to the decision of the Breast Cancer Committee of our institution and were treated with four cycles of FEC. After chemotherapy, surgery was performed in all 86 patients. Mastectomy was performed in 78 patients (91%) and lumpectomy in eight (9%) according to the decision of the Breast Cancer Committee of each institution. Pathological stages after surgery were as follows: one patient (1%) was stage 0; eight (9%) were stage I, 32 (37%) were stage II, 45 (53%) were stage III. At the time of surgery, 69 patients (80%) had nodal involvement and 17 (20%) did not. The median number of involved nodes was 3 (range 0-26). Seventy-nine patients (92%) had ductal carcinoma, five (6%) had lobular carcinoma and two (2%) had other histologies (mucinous, medular) (Table 1). ER, PR, CK 5/6, HER2, and Vimentin status are shown in Table 1. Median follow-up for all patients was 50.5 months (range, 5-154), and median follow-up for alive patients was 77 months (range, 40–154). RT-QPCR and tissue microarray analyses were performed in the surgical specimens, except for one patient who presented a pathologic complete response, in whom analyses were performed in the pretreatment biopsy.

**Table 1:** Patient characteristics.

	Entire Cohort (N = 86) N (%)	Patients without BRCA1 Assessment (N = 45) N (%)	Patients with BRCA1 Assessment (N = 41) N (%)	p
**Age, median (range)**	54 (31–79)	56 (34–74)	55 (31–79)	0.78
**Menopausal status**				0.51
** Premenopausal**	36 (42%)	28 (62.2)	22 (54%)	
** Postmenopausal**	50 (58%)	17 (37.8)	19 (46%)	
**Tumor size (cm)**	6 (2.5–12)	6 (2.50–12)	6.4 (2.5–12)	0.34
**Tumor differentiation**				0.52
** Grade I**	8 (10.4)	5 (13.2)	3 (7.7)	
** Grade II**	34 (44.2)	18 (47.4)	16 (41)	
** Grade III**	35 (45.5)	15 (39.5)	20 (51.3)	
** Unknown**	9	7	2	
**Clinical stage**				0.51
** II**	24 (28%)	14 (31.1)	10 (24%)	
** III**	62 (72%)	31 (68.9)	31 (76%)	
**Pathological response**				0.63
** Response (G5, G4, G3)**	49 (57%)	27 (60)	22 (53.7%)	
** No response (G2, G1)**	37 (43%)	18 (40)	19 (46.3%)	
**Surgery**				0.54
** Mastectomy**	78 (91%)	39 (86.6)	39 (95%)	
** Lumpectomy**	8 (9%)	6 (13.3)	2 (5%)	
**Pathological nodal status**				0.29
** Positive**	69 (80%)	34 (75.6)	35 (85%)	
** Negative**	17 (20%)	11 (24.4)	6 (15%)	
**Histology**				0.41
** Invasive ductal carcinoma**	79 (92%)	41 (91.1)	38 (93%)	
** Invasive lobular carcinoma**	5 (6%)	3 (6.7)	2 (5%)	
** Other: mucinous, medular**	2 (2%)	1 (2.2)	1 (2%)	
**Estrogen receptor**				0.38
** 0–4%**	35 (40.7)	16 (35.6)	19 (46.3)	
** 5–100%**	51 (59.3)	29 (64.4)	22 (53.7)	
**Progesterone receptor**				0.65
** 0–4%**	59 (68.6)	32 (71.1)	27 (65.9)	
** 5–100%**	27 (31.4)	13 (28.9)	14 (34.1)	
**Cytokeratin 5/6** *				0.60
** Negative**	66 (78.6)	35 (81.4)	31 (75.6)	
** Positive**	18 (21.4)	8 (18.6)	10 (24.4)	
**HER2 by CISH**				0.99
** Positive**	17 (20.2)	9 (20.5)	8 (20)	
** Negative**	67 (79.8)	35 (79.5)	32 (80)	
**Vimentin** *				0.56
** Negative**	72 (84.7)	37 (82.2)	35 (87.5)	
** Positive**	13 (15.3)	8 (17.8)	5 (12.5)	
**Subtypes** *				0.73
** HER2+/ER-**	11 (13.1)	5 (11.4)	6 (15)	
** Luminal A**	45 (53.6)	25 (56.8)	20 (50)	
** Luminal B**	6 (7.1)	4 (9.1)	2 (5)	
**Basal-like**	22 (26.2)	10 (22.7)	12 (30)	
**BRCA1 (Median, range)**	----	----	16.68 (2.93–187.40)	----

Clinical characteristics and results of molecular analyses for all 86 patients, for the 41 patients in whom BRCA1 mRNA expression was assessed and for the 45 in whom BRCA1mRNA was not assessed.

*Technical issues made it impossible to assess some patients.

### Response, TTP and OS

Pathological response was evaluated according to the Miller and Payne criteria [21], as shown in Table 2. In one case (1.2%), the pathological remission was ganglionar and locally complete (G5); in 44 cases (51.2%), there were signs of inflammation, necrosis and fibrosis with the presence of local infiltrating ductal carcinoma nests (G4, G3); in 37 cases (43%), no evidence of histological response was observed (G2, G1). Median TTP was 42 months (95% CI, 21.66–62.34), and median OS was 55 months (95%CI, 30.09–79.91).

**Table 2:** Patient Characteristics.

	Entire Cohort			Patients with BRCA1 Assesment		
	(N = 86)			(N = 41)		
RESPONSE	Local	Axilla*	Local + Axilla	Local	Axilla+	Local + Axilla
CR G5	5 (5.8%)	D	1 (1.2%)	1 (2.5%)	C	1 (2.4%)
		C	4 (4.7%)			
PR G4+G3	44 (51.2%)	D	6 (6.9%)	21 (51.2%)	D	2 (4.9%)
		C+B	32 (37.2%)		C+B	16 (39.0%)
**NO RESPONSE**						
G1+G2	37 (43%)	C+B	33 (38.4%)	19 (46.3%)	D	1 (2.4%)
					C+B	18 (44%)

Detailed pathologic response for the entire cohort and for the patients with BRCA1 assessment, according to the Miller and Payne criteria.

CR  =  complete response; PR  =  partial response. Pathologic response assessment according to the Miller and Payne classification. G5: absence of residual infiltrating tumour cells. G4: marked disappearance of invasive tumor cells, only small clusters of widely dispersed cells could be detected. G3: considerable reduction in tumor cells. G2: mild loss of invasive tumor cells, but overall cellularity still high. G1: no reduction in overall numbers as compared with pre-treatment biopsy. B: lymph node positive with malignant cells. C: lymph node still positive, but with evidence of some regression. D: lymph node previously positive, now without metastases.

* In a total of 10 patientes (11.6%) axilla lymphadenectomy was negative but was also clinically negative before surgery (6 response and 4 no response).

+ In a total of 3 patientes (7.3%) axilla lymphadenectomy was negative but was also clinically negative before surgery (2 response and 1 no response).

### Immunohistochemical Analysis and Outcomes

Based on ER, PR, and HER2 status, 45 patients (52%) were classified as luminal A, six (7%) as luminal B, 13 (15%) as HER2+/ER-, and 22 (26%) as basal-like.

CK5/6 were expressed in 18 tumors (21.4%), of which 11 (61.1%) were basal-like, four (22.2%) HER2+/ ER-, two (11.1%) was luminal A, and one (5.6%) was luminal B. Thirteen tumors (15.3%) expressed vimentin, of which ten (76.9%) were basal-like, two (15.4%) were HER2+/ ER-, and one (7.7%) was luminal B. Since only one patient attained a pathological complete response, it is impossible to correlate response with any of the potential markers.

In the univariate analyses for TTP and OS for all 86 patients, those with ER-negative tumors had a higher risk of relapse (HR, 2.25; p = 0.005) and death (HR, 2.51; p = 0.002) than those with ER-positive tumors. When patients were grouped according to subtypes, basal-like patients had the worse prognosis (Table 3). In the multivariate analyses for TTP and OS, ER-negative was again identified as a predictive variable (Table 3).

**Table 3:** Univariate analyses for time to progression and overall survival for all 86 patients and for 41 patients in whom BRCA1 was assessed.

	All Patients (N = 86)					Patients with BRCA1 Assessment (N = 41)				
		Time to Progression		Overall Survival			Time to Progression		Overall Survival	
	N	HR (95% CI)	P	HR (95% CI)	P	N	HR (95% CI)	P	HR (95% CI)	p
**BRCA1 by terciles**										
** Low**						14	1		1	
** Intermediate**	---	---	---	---	---	14	4.55 (1.61–12.85)	**0.004**	3.90 (1.34–11.38)	**0.01**
** High**						13	2.42 (0.87–6.76)	0.09	2.41 (0.82–7.09)	0.11
**Estrogen receptor**										
** Negative**	35	2.25 (1.28–3.95)	**0.005**	2.51 (1.41–4.47)	**0.002**	19	1.57 (0.73–3.36)	0.25	1.70 (0.78–3.69)	0.18
** Positive**	51	1		1		22	1		1	
**Progesterone receptor**										
** Negative**	59	1.40 (0.76–2.58)	0.28	1.74 (0.90–3.36)	0.10	27	1.80 (0.77–4.19)	0.17	1.98 (0.83–4.74)	0.13
** Positive**	27	1		1		14	1		1	
**HER2 by CISH**										0.30
** Negative**	67	1	0.35	1	0.22	32	1	0.34	1	
** Positive**	17	1.38 (0.70–2.71)		1.53 (0.78–3.03)		8	1.57 (0.62–3.96)		1.63 (0.65–4.12)	
**Nodal status**										
** Negative**	17	1	0.06	1	0.11	6	1	**0.05**	1	0.19
** Positive**	69	2.25 (0.95–5.28)		2.03 (0.86–4.78)		35	7.52 (0.99–56.75)		2.61 (0.61–11.15)	
**Vimentin**										
** Negative**	72	1.26 (0.54–2.97)	0.59	1.01 (0.45–2.23)	0.99	35	1.13 (0.34–3.77)	0.85	0.65 (0.22–1.88)	0.42
** Positive**	13	1		1		5	1		1	
**Cytokeratin 5/6**										
** Negative**	66	1.06 (0.51–2.18)	0.88	0.83 (0.41–1.67)	0.59	31	1.28 (0.48–3.39)	0.62	0.90 (0.36–2.26)	0.83
** Positive**	18	1		1		10	1		1	
**Subtypes**										
** HER2+/ER-**	11	0.73 (0.30–1.79)	0.49	0.92 (0.38–2.26)	0.86	6	0.88 (0.26–2.95)	0.84	0.97 (0.30–3.18)	0.96
** Luminal A**	45	0.39 (0.20–0.75)	**0.005**	0.38 (0.19–0.74)	**0.005**	20	0.56 (0.23–1.38)	0.21	0.54 (0.22–1.34)	0.18
** Luminal B**	6	0.74 (0.25–2.21)	0.59	0.69 (0.23–2.06)	0.50	2	2.07 (0.43–10.05)	0.37	1.65 (0.35–7.82)	0.53
** Basal-like**	22	1		1		12	1		1	
**Age**	86	1.02 (0.99–1.04)	0.23	1.01 (0.99–1.04)	0.29	41	1.02 (0.99–1.05)	0.28	1.01 (0.98–1.05)	0.38

### BRCA1 mRNA Expression and Outcomes

Median BRCA1 mRNA expression in the 41 samples assessed was 16.68 (range, 2.93–187.40). When we compared the results of the two additional housekeeping genes (r18S, RPLP0) with those obtained in the original analysis using β-actin, using the Spearman correlation test (two-sided), a significant correlation among the three genes was observed: β-actin vs r18S (ρ = 0.70; p<0,001); β-actin vs RPLP0 (ρ = 0.61; p<0.001); RPLP0 vs r18S (ρ = 0.77; p<0.001). There was also a significant correlation of BRCA1 expression data calculated according to each of the three housekeeping genes: BRCA1/β-actin vs BRCA1/β-actin plus RPLP0 (ρ = 0.60; p<0.001); BRCA1/β-actin vs BRCA1/β-actin plus r18S (ρ = 0.70; p<0,001) (Supplementary Table S1).

**Figure 1:**
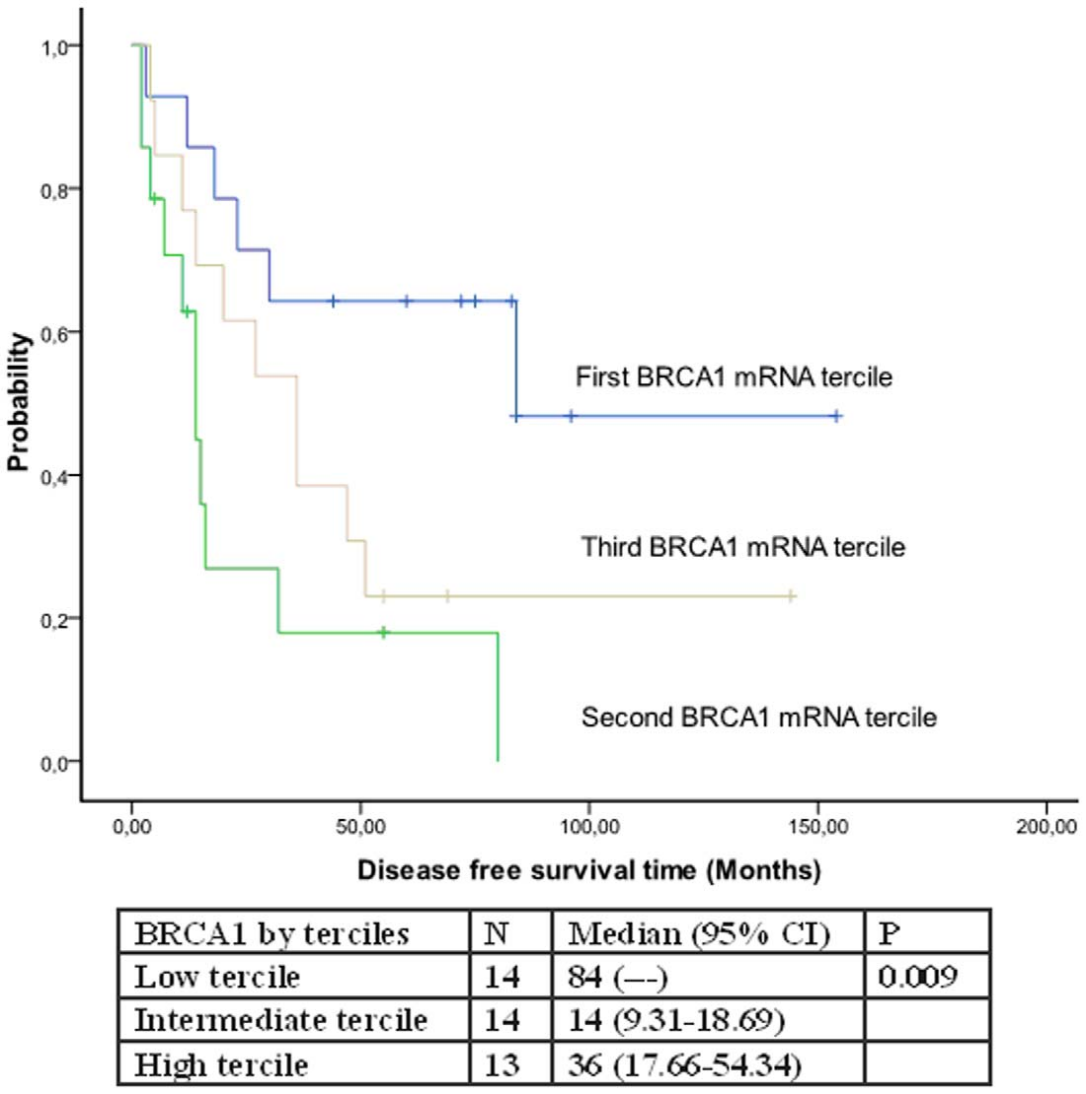
Time to progression according to BRCA1 mRNA levels.

**Figure 2:**
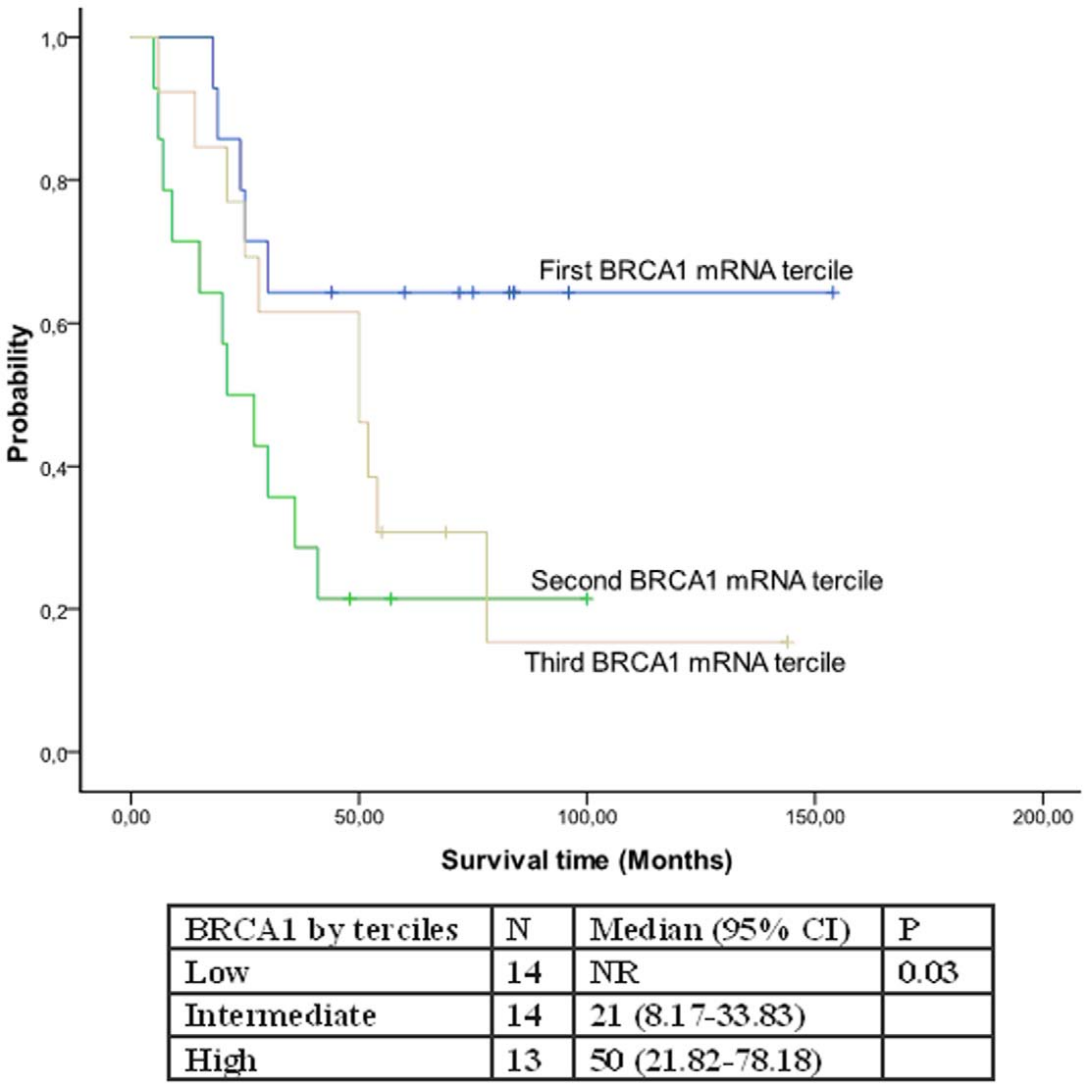
Overall survival according to BRCA1 mRNA levels.

There were no differences in patient characteristics according to BRCA1 mRNA levels (Table 4). Only one patient attained a pathological complete response (in tumor but not in axilla); it is thus impossible to correlate response with BRCA1 mRNA levels. Low levels of BRCA1 mRNA were associated with better TTP (84 months versus 14 and 36 months; p = 0.009) (Figure 1). Median OS was not reached in patients with low levels of BRCA1 mRNA, while it was 21 months for those with intermediate and 50 months for those with high levels (p = 0.03) (Figure 2).

**Table 4:** Patient characteristics in 41 patients according to BRCA1 mRNA levels by terciles.

	Low BRCA1	Intermediate BRCA1	High BRCA1	p
	N (%)	N (%)	N (%)	
**Age, median (range)**	59 (45–73)	51 (32–79)	54 (31–74)	0.25
**Menopausal status**				0.52
** Premenopausal**	5 (26.3)	8 (42.1)	6 (31.6)	
** Postmenopausal**	9 (40.9)	6 (27.3)	7 (31.8)	
**Tumor size (cm)**	6 (4–10)	7 (2.50–12)	6 (4–11)	0.60
**Tumor differentiation**				0.56
** Grade I**	0	2 (14.3)	1 (8.3)	
** Grade II**	6 (46.2)	4 (28.6)	6 (50)	
** Grade III**	7 (53.8)	8 (57.1)	5 (41.7)	
**Pathological Response**				0.26
** Response (G5, G4, G3)**	10 (45.5)	6 (27.3)	6 (27.3)	
** No response (G2, G1)**	4 (21.1)	8 (42.1)	7 (36.8)	
**Pathological Nodal status**				0.99
** Negative**	2 (33.3)	2 (33.3)	2 (33.3)	
** Positive**	12 (34.3)	12 (34.3)	11 (31.4)	
**Cytokeratin 5/6**				0.10
** Negative**	11 (35.5)	8 (25.8)	12 (38.7)	
** Positive**	3 (30)	6 (60)	1 (10)	
**HER2 by CISH**				0.43
** Positive**	3 (37.5)	4 (50)	1 (12.5)	
** Negative**	11 (34.4)	10 (31.3)	11 (34.4)	
**Estrogen receptor**				0.79
** Negative (0–4%)**	7 (36.8)	7 (36.8)	5 (26.3)	
** Positive (5–100%)**	7 (31.8)	7 (31.8)	8 (36.4)	
**Progesterone receptor**				0.18
** Negative (0–4%)**	10 (37)	11 (40.7)	6 (22.2)	
** Positive (5–100%)**	4 (28.6)	3 (21.4)	7 (50)	
**Vimentin**				0.74
** Negative**	13 (37.1)	12 (34.3)	10 (28.6)	
** Positive**	1 (20)	2 (40)	2 (40)	
**Subtypes**				0.43
** HER2+/ER-**	3 (50)	2 (33.3)	1 (16.7)	
** Luminal A**	7 (35)	5 (25)	8 (40)	
** Luminal B**	0	2 (100)	0	
** Basal-like**	4 (33.3)	5 (41.7)	3 (25)	

In the univariate analysis for TTP and OS for these 41 patients, low levels of BRCA1 mRNA were associated with a lower risk of relapse and longer survival (p = 0.004 and p = 0.01, respectively), and positive nodal status was associated with shorter TTP (p = 0.005) (Table 3). In the multivariate analysis for TTP and OS, low levels of BRCA1 mRNA, PR+, and negative lymph node involvement predicted a lower risk of relapse, while low levels of BRCA1 mRNA and PR+ were the only variables associated with significantly better survival (Table 5).

**Table 5:** Multivariate analyses for time to progression and overall survival in 41 patients with BRCA assessment.

		Time to Progression		Overall Survival	
	N	HR (95% CI)	P	HR (95% CI)	P
**BRCA1 by terciles**					
** Low**	14	1		1	
** Intermediate**	14	7.68 (2.41–24.55)	0.001	4.52 (1.51–13.46)	0.007
** High**	13	4.52 (1.50–13.63)	0.007	2.94 (0.99–8.71)	0.05
**Progesterone receptor**					
** Negative**	27	4.15 (1.61–10.69)	0.003	2.43 (0.99–5.93)	0.05
** Positive**	14	1		1	
**Nodal Status**				--------	
** Negative**	6	1	0.02		
** Positive**	35	12.01 (1.51–95.16)			

## Discussion

Patients were treated with a schedule of only four cycles of anthracyclines before surgery, which was standard practice at the time of the study, before taxanes were introduced in the primary treatment setting; this could explain the relatively low rate of pathological complete responses (1.2%) and conservative surgery (8%). Current schedules of neoadjuvant chemotherapy, with six to eight cycles of sequential chemotherapy with anthracyclines and taxanes, attain an improved rate of pathological complete responses (20–30%) and conservative surgery (61–67%) [22], [23].

Similarly to findings by Sorlie et al [5], in our series, patients with luminal A tumors attained better TTP and OS than those with basal-like tumors**.** In the multivariate analysis including all 86 patients, ER emerged as the strongest predictor of TTP and OS. Vimentin expression is associated with high tumor invasiveness; in our cohort, vimentin expression was found only in basal-like and in HER2+/ER- tumors.

In the multivariate analysis of the subset of 41 patients, BRCA1 mRNA expression level emerged as the strongest predictor of survival. BRCA1 plays a crucial role in DNA repair and decreased BRCA1 mRNA has been observed in both sporadic and hereditary breast cancer. BRCA1 mRNA is reduced in sporadic breast cancer cells despite the absence of mutations. This reduction of BRCA1 mRNA levels in sporadic breast cancer cases has been related to acquired methylation of the BRCA1 promoter [24] and to abnormalities in the upstream pathways that regulate BRCA1 expression[25].

BRCA1 encodes a nuclear cell cycle regulated protein expressed in S and G2 phases, which may be why BRCA1 overexpression has been associated with poor survival in chemonaive NSCLC[26]. In sporadic breast cancer, absent or reduced BRCA1 expression was associated with high tumor grade, advanced lymph node stage, larger size, vascular invasion, negative estrogen receptor, negative progesterone receptor and poor outcome[27]. BRCA1 plays a multifunctional role and has been implicated in many normal cellular functions, including DNA damage response, transcriptional regulation, cell-cycle checkpoint control, and ubiquitination. Consequently, the presence or absence of functional BRCA1 could have a significant effect on cellular response to chemotherapy and may also have a predictive value, particularly in patients treated with DNA-damaging agents, as was the case in the present study.

Preclinical data suggest that BRCA1 can regulate differential sensitivity to chemotherapeutic agents; the absence of BRCA1 results in increased sensitivity to DNA-damage-based chemotherapy, while the presence of BRCA1 increases sensitivity to antimicrotubule agents. It was initially reported that BRCA1 overexpression in human breast cancer cell lines resulted in increased resistance to DNA-damaging chemotherapy [28], [29], [30]. In HCC1937 cells, restoring BRCA1 abrogated sensitivity to apoptosis in the presence of DNA-damaging agents, including cisplatin and etoposide, while inducing sensitivity to the antimicrotubule agents paclitaxel and vinorelbine, suggesting that BRCA1 acts as a differential modulator of apoptosis depending on the nature of the cellular insult [31]. In a recent report, overexpression of BRCA1 and other genes (p53, p21, GST, MDR1 and TOPOIIα) have been associated with acquired resistance to doxorubicin in breast cancer cell lines [32].

A differential modulating effect for BRCA1 mRNA expression was also observed in tumor cells isolated from malignant effusions of non-small-cell lung cancer and gastric cancer patients, whose BRCA1 mRNA levels correlated negatively with cisplatin sensitivity and positively with docetaxel sensitivity [33]. In addition, several clinical studies have shown a better clinical response to anthracycline- and cyclophosphamide- containing regimens in BRCA1 mutation carriers than in sporadic breast cancer patients [17]. Upregulation of DNA repair genes has been related to resistance to radiotherapy, and BRCA1 mutation carriers are more sensitive to radiotherapy [34].

In sporadic breast cancer cases, there is conflicting evidence as to whether tumors with epigenetic inactivation of BRCA1 will also obtain greater benefit from DNA-damage-based chemotherapy. In a study of 51 sporadic breast cancer patients, those with high levels of BRCA1 attained better response to anthracycline-based chemotherapy, though overall survival was not examined [34]. This result is in contrast with results from *in vitro* models, BRCA1 mutated breast cancers, and our results in the present study.

In a retrospective cohort of 70 sporadic epithelial ovarian cancer patients treated with platinum-based chemotherapy, those with low levels of BRCA1 mRNA expression had a significantly improved survival in comparison with those with high levels [35]. Furthermore, BRCA1 mRNA expression levels predicted outcome following cisplatin-containing chemotherapy in non-small-cell lung cancer [18]. Along the same lines, in a retrospective study of locally advanced bladder cancer patients treated with cisplatin-based chemotherapy, those with low or intermediate levels of BRCA1 mRNA attained significantly better response, disease-free survival and OS than those with high levels[36].

In a recent series of 102 non-small-cell lung cancer patients, high levels of BRCA1 mRNA were associated with better response and decreased risk of progression to gemcitabine plus docetaxel [37]. Our findings are consistent with this clinical evidence and with other clinical studies in breast cancer. In a study comparing BRCA1 germ-line mutation carriers and non-carriers, response rates to neoadjuvant docetaxel treatment in the carrier group was limited while non-carriers attained a high number of complete or partial responses [38]. All these data suggest that BRCA1 plays an important role in cellular response to chemotherapy, not only to DNA damaging agents but also to antimicrotubules. In fact, in a recent study in which patients with low BRCA1 mRNA levels were treated with cisplatin and gemcitabine, those with intermediate levels were treated with cisplatin and docetaxel, and patients with high levels were treated with docetaxel alone, this BRCA1-customized chemotherapy was associated with excellent 2-year survival for patients with metastatic non-squamous cell lung carcinoma[39].

We provide clinical evidence in 41 patients for the role of post-treatment BRCA1 mRNA levels as a marker of TTP and OS in in sporadic breast cancer patients treated with anthacyclines. Although our findings should be interpreted with caution due to the small number of patients and the retrospective nature of the study, they warrant further examination in prospective clinical trials including pre-treatment BRCA1 assessment. Our data suggest that sporadic breast cancer patients with low levels of BRCA1 mRNA expression may obtain the greatest benefit from anthracycline-based therapy.

## Materials and Methods

### Patients

We collected tumor biopsies from 86 patients diagnosed with stage II and stage III breast cancer who were who were not eligible for conservative breast treatment and were considered to be candidates for primary therapy according to the decision of the Breast Cancer Committee of our institution between 1993 and 2003. The study was approved by the Ethics Committee of our institution. Signed informed consent for future biological studies of tumor biopsies was obtained from all patients. All patients received four cycles of FEC before surgery. After surgery, patients were evaluated and received four additional cycles of the same schedule if they had no nodal involvement and six if they had nodal involvement. If the tumor was ER- or PR-positive, they received hormonotherapy after completion of chemotherapy. Patients undergoing lumpectomy also received breast radiotherapy, and regional nodal radiation was delivered at the discretion of the breast committee. Samples were collected and evaluated at the Catalan Institute of Oncology (Badalona, Barcelona, Spain). Pathological response was evaluated according to the Miller and Payne criteria [21]. Patients without residual infiltrating cancer in the breast and axilla were considered to have had a pathological complete response.

### Immunohistochemical Analysis

Breast cancer tissue microarrays were selected based on the availability of paraffin blocks and prepared by extracting two 1-mm diameter cores of histological confirmed invasive breast carcinoma and immunohistochemically stained for ER, PR, basal cytokeratins HER2, CK5/6 and vimentin. Two cores were evaluated from each tumor. Each core was scored individually, and the mean of the two readings was calculated.

ER and PR were considered positive only if nuclear positivity was seen in more than 5% of neoplastic cells. HER2 staining was scored according to the criteria specified by DAKO for the interpretation of the HercepTest. Immunoreaction was determined to be strongly positive (3+) if a strong complete membrane staining was observed in more than 10% of neoplastic cells or to be weakly positive (2+) if more than 10% of the tumor cells showed weak to moderate complete membrane staining. All other staining patterns were interpreted as negative (0/1+). Chromogenic *in situ* hybridization (CISH) was performed on HER2 immunopositive cases (2+ or 3+) using a microscope (Leica DMLS2) equipped with 10X, 20X, 40X and 63X dry objectives, with 10x oculars. The revised standard described by Tanner et al [40], was used to interpret CISH results.

CK 5/6 and vimentin staining results were assessed using a three-point scoring system, where 0 was no staining, 1 was weak staining intensity and/or less than 20% of tumor cells stained, and 2 was strong staining in more than 20% of tumor cells.

### BRCA1mRNA Expression

**Figure 3:**
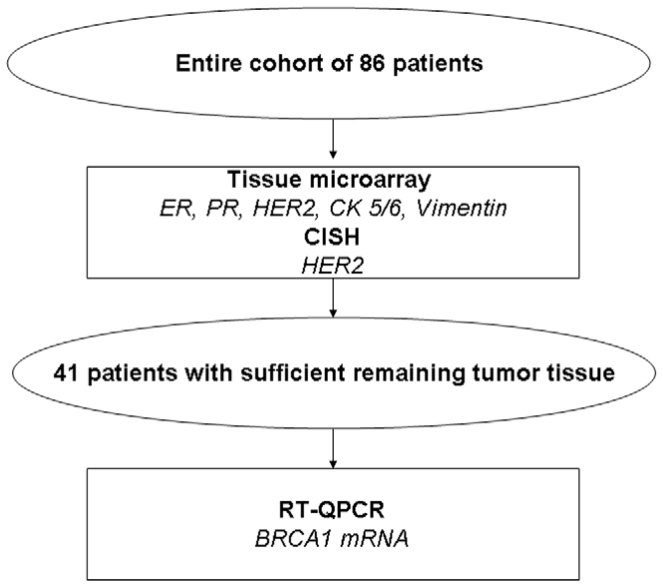
Flow chart of patients. Of 86 patients originally included, only 41 had sufficient tumor tissue to perform BRCA1 mRNA assessment.

Intratumoral BRCA1 mRNA expression levels were assessed by RT-QPCR in 41 patients for whom sufficient tumor tissue was available (Fig. 3). Total RNA was extracted from paraffin-embedded tumor tissue after laser-capture microdissection that ensured a minimum of 90% of tumor cells. After deparaffinization with standard xylene and alcohol process, samples were subjected to lysis in a buffer containing tris-chloride, EDTA, sodium dodecyl sulphate, and proteinase K. RNA was then extracted with phenol-chlorophorm-isoamyl alcohol followed by precipitation with isopropanolol in the presence of glycogen and sodium acetate. RNA was resuspended in DEPC water (Ambion Inc., Austin, TX) and treated with DNAse I to avoid DNA contamination. cDNA was synthesized using M-MLV retrotranscriptase enzyme. Template cDNA was added to TaqMan Universal Master Mix in a 12.5-µL reaction with specific primers and probe for each gene. The endogenous reference gene was β-actin. Gene expression was quantified by using the ABI Prism 7900HT Sequence Detection System (Applied Biosystems)[18]. In order to preclude any potential false results due to variation in RNA quality in paraffin-embedded samples and also to validate the robustness of β-actin as a single housekeeping gene, in a subgroup of 34 patients for whom RNA was available, BRCA1 expression data was also calculated according to the median values of β-actin plus RPLP0 and β-actin plus ribosomal 18S as housekeeping genes.

### Statistical Analyses

Median values and ranges were derived for quantitative variables and mRNA gene expression. Qualitative variables were summarized by means of absolute frequencies and percentages. In order to provide an easily interpretable evaluation of the effect of BRCA1 mRNA expression, gene expression values were divided into terciles.

TTP was calculated from the time of inclusion in the study until disease progression. Overall survival was estimated from the time of inclusion until death from any cause. TTP and OS were calculated using Kaplan-Meier estimates and differences between curves were tested using the log-rank test. The Cox proportional hazards method with hazard ratios and 95% confidence intervals (CIs) were used to fit both univariate and multivariate models, where stepwise procedure (both forward and backward) were used to evaluate the independent significance of different variables in TTP and OS. Analyses were performed using Statistical Package for the Social Sciences (SPSS) for Windows version 17 (SPSS Inc, Chicago, IL) and S-Plus 6.1 for Windows.

## Supporting Information

Table S1 RNA for additional analyses was available in 34 of the original 41 samples, and BRCA1 gene expression was assessed in these 34 samples using two additional housekeeping genes, ribosomal 18S (r18s) and RPLP0. A significant correlation among the three genes was observed (p<0.001). Data from real-time QPCR is shown here.
(0.08 MB DOC)
