# Supplementary material for: The Prognostic Value of BRCA1 mRNA Expression Levels Following Neoadjuvant Chemotherapy in Breast Cancer
Source: PLoS One. 2010 Mar 3;5(3):e9499. doi: 10.1371/journal.pone.0009499 (PMC2831058; doi:10.1371/journal.pone.0009499)
Supplement: Table S1 — RNA for additional analyses was available in 34 of the original 41 samples, and BRCA1 gene expression was assessed in these 34 samples using two additional housekeeping genes, ribosomal 18S (r18s) and RPLP0. A significant correlation among the three genes was observed (p<0.001). Data from real-time QPCR is shown here. (0.08 MB DOC) [file pone.0009499.s001.doc]

**Supplementary Table 1.** RNA for additional analyses was available in 34 of the original 41 samples, and BRCA1 gene expression was assessed in these 34 samples using two additional housekeeping genes, ribosomal 18S (r18s) and RPLP0. A significant correlation among the three genes was observed (p<0.001). Data from real-time QPCR is shown here.

|  |  |  |  | **BRCA1 RNA expression** | |
| --- | --- | --- | --- | --- | --- |
|  |  |  |  |
| **Number** | **Ct 18S** | **Ct RPLP0** | **Ct β-actin** | **2^-ΔΔCt^ BRCA1 vs β-act/18S** | **2^-ΔΔCt^ BRCA1 vs β-act/RPLP0** |
| **NM 2** | 21.37 | 33.22 | 30.79 | 40.79 | 13.50 |
| **NM 3** | 17.45 | 30.43 | 26.53 | 12.21 | 5.98 |
| **NM 4** | 21.15 | 32.49 | 31.93 | 59.30 | 16.45 |
| **NM 6** | 19.84 | 32.06 | 27.87 | 15.30 | 5.76 |
| **NM 7** | 19.81 | 31.39 | 31.80 | 77.44 | 23.34 |
| **NM 11** | 17.72 | 30.78 | 26.34 | 9.85 | 4.96 |
| **NM 12** | 16.44 | 28.81 | 24.60 | 8.06 | 3.19 |
| **NM 13** | 20.50 | 33.2 | 29.99 | 18.96 | 8.43 |
| **NM 14** | 18.21 | 29.96 | 28.52 | 13.41 | 4.29 |
| **NM 15** | 17.86 | 30.46 | 28.79 | 11.04 | 4.74 |
| **NM 16** | 19.55 | 33.11 | 30.45 | 20.11 | 12.04 |
| **NM 20** | 18.27 | 30.86 | 28.82 | 18.96 | 8.11 |
| **NM 33** | 18.12 | 31.09 | 27.94 | 9.06 | 4.42 |
| **NM 34** | 20.51 | 32.86 | 28.41 | 30.06 | 11.84 |
| **NM 36** | 18.53 | 31.46 | 27.42 | 5.41 | 2.60 |
| **NM 37** | 18.92 | 33.32 | 33.00 | 40.22 | 32.22 |
| **NM 38** | 19.00 | 31.82 | 27.51 | 23.83 | 11.04 |
| **NM 40** | 17.93 | 32.34 | 29.78 | 9.75 | 7.84 |
| **NM 41** | 19.23 | 32.36 | 28.80 | 22.39 | 11.55 |
| **NM 42** | 19.67 | 31.91 | 29.38 | 13.98 | 5.30 |
| **NM 43** | 17.00 | 30.22 | 26.72 | 8.69 | 4.63 |
| **NM 46** | 19.41 | 33.25 | 27.99 | 7.52 | 4.96 |
| **NM 47** | 19.18 | 32.08 | 27.70 | 3.01 | 1.43 |
| **NM 48** | 19.40 | 33.36 | 29.93 | 25.90 | 17.81 |
| **NM 49** | 19.33 | 31.14 | 28.48 | 9.55 | 3.12 |
| **NM 50** | 17.95 | 30.4 | 28.72 | 6.89 | 2.81 |
| **NM 52** | 18.28 | 31.31 | 27.24 | 15.56 | 7.75 |
| **NM 53** | 18.49 | 32.75 | 28.33 | 6.11 | 4.66 |
| **NM 64** | 16.98 | 30.27 | 25.52 | 10.27 | 5.60 |
| **NM 66** | 21.23 | 33.95 | 28.89 | 10.41 | 4.66 |
| **NM 67** | 18.13 | 31.26 | 26.69 | 13.18 | 6.80 |
| **NM 68** | 17.80 | 31.18 | 27.83 | 20.04 | 11.27 |
| **NM 69** | 19.63 | 32.22 | 28.59 | 42.22 | 18.06 |
| **NM 70** | 18.68 | 33.51 | 28.34 | 11.39 | 10.59 |
